# Clinical observation of laparoscopic sleeve gastrectomy and metformin treatment in obese PCOS patients

**DOI:** 10.5937/jomb0-44411

**Published:** 2024-04-23

**Authors:** Qingya Ma, Xiaojing He, Zijie Fu, Xiaodong Ren, Ranran Sun, Siqi Zhu, Yahui Bian, Xiaodong Li

**Affiliations:** 1 The First Hospital of Hebei Medical University, Department of Gynecology, Shijiazhuang, China; 2 The Second Hospital of Hebei Medical University, Department of Obstetrics and Gynaecology, Shijiazhuang, China

**Keywords:** PCOS, obesity, laparoscopic sleeve gastrectomy, metformin, PCOS, gojaznost, laparoskopska rukavna gastrektomija, metformin

## Abstract

**Background:**

To observe the basic metabolic characteristics of obese patients with polycystic ovarian syndrome (PCOS), and observe and compare the effect of laparoscopic sleeve gastrectomy and metformin treatment after 3 months.

**Methods:**

In January to December 2018, the Second Hospital of Hebei Medical University selected 104 women who were classified as obese with a body mass index (BMI) of 28 kg/cm2 or higher and had PCOS. They were divided into obese PCOS group (53 cases) and obese non-PCOS group (51 cases).

**Results:**

1. There was no significant difference in waist circumference and WHR between patients who are obese with PCOS and patients who are obese without PCOS (P > 0.05). Obese PCOS patients were significantly higher in anti-Müllerian hormone (AMH), LH/FSH, T, FAI, homa-ir, triglyceride (TG), low density lipoprotein (LDL), Apo-B and uric acid than the group of non-PCOS patients who were obese. (P<0.05). The SHBG levels of obese patients with PCOS were obviously lower when contrasted with the levels in obese patients without PCOS (P < 0.05). 2. Body weight, BMI, INS, homa-ir and TG of obese PCOS patients were significantly decreased 3 months after laparoscopic sleeve gastrectomy compared with that before surgery (P < 0.05). After three months of medical treatment with metformin, the patients' homeostatic model assessment of insulin resistance (HOMA-IR) was obviously reduced when contrasted with the pre-treatment HOMA-IR levels (P < 0.05), and there was no significant difference in the improvement degree of homa-ir between the two groups (P > 0.05).

**Conclusions:**

1. Obese patients with PCOS demonstrated higher expression of AMH, LH/FSH, T, SHBG, and FAI when contrasted with the control group. Additionally, they experienced more severe insulin resistance and lipid metabolism disorders. 2. The weight and BMI of obese PCOS patients were significantly decreased after weight loss, while IR and blood lipid were significantly improved, while IR was improved in metformin group, and no significant discrepancy was observed in the degree of improvement of insulin resistance between both groups.

## Abstract (sr)

**Uvod:**

Cilj je bio da se sagledaju osnovne metaboličke karakteristike gojaznih pacijenata sa sindromom policističnih jajnika (PCOS), i posmatra i uporedi efekat laparoskopske sleeve gastrektomije i lečenja metforminom posle 3 meseca.

**Metode:**

U periodu od januara do decembra 2018. Druga bolnica Medicinskog univerziteta Hebei odabrala je 104 žene koje su klasifikovane kao gojazne sa indeksom telesne mase (BMI) od 28 kg/cm 2 ili više i koje su imale PCOS. Oni su podeljeni u gojaznu grupu sa PCOS (53 slučaja) i gojaznu grupu bez PCOS (51 slučaj).

**Rezultati:**

1. Nije bilo značajne razlike u obimu struka i VHR između pacijenata koji su gojazni sa PCOS i pacijenata koji su gojazni bez PCOS (P > 0,05). Gojazni pacijenti sa PCOS su bili značajno viši u anti-Mullerian hormonu (AMH), LH /FSH, T, FAI, homair, triglicerid (TG), lipoprotein niske gustine (LDL), Apo-B i mokraćna kiselina u odnosu na grupu pacijenata bez PCOS koji su bili gojazni. (P<0,05). Nivoi SHBG kod gojaznih pacijenata sa PCOS su očigledno bili niži u poređenju sa nivoima kod gojaznih pacijenata bez PCOS (P <0,05). 2. Telesna težina, BMI, INS, homa-ir i TG gojaznih pacijenata sa PCOS su značajno smanjeni 3 meseca nakon laparoskopske rukavne gastrektomije u poređenju sa onim pre operacije (P < 0,05). Nakon tri meseca lečenja metforminom, homeostatska procena insulinske rezistencije (HOMA-IR) pacijenata je očigledno smanjena u poređenju sa nivoima HOMA-IR pre tretmana (P < 0,05), i nije bilo značajne razlike u stepen poboljšanja homa-ir izme|u dve grupe (P > 0,05).

**Zaključak:**

1. Gojazni pacijenti sa PCOS su pokazali veću ekspresiju AMH, LH/FSH, T, SHBG i FAI u poređenju sa kontrolnom grupom. Pored toga, iskusili su ozbiljniju insulinsku rezistenciju i poremećaje metabolizma lipida. 2. Težina i BMI gojaznih pacijenata sa PCOS su znaćajno smanjeni nakon gubitka težine, dok su IR i lipid u krvi značajno poboljšani, dok je IR poboljšan u metformin grupi i nije uočena značajna razlika u stepenu poboljšanja insulinske rezistencije između oba. grupe.

## Introduction

The last four decades have seen a global increase in obesity rates, which has been paralleled by an escalation in the incidence of co-morbidities linked to obesity, including polycystic ovary syndrome (PCOS). PCOS is an endocrine disorder with a prevalence of about 5%–10% [1], and is characterized by menstrual disorders, infertility, acne vulgaris, and polycystic ovarian changes. Laboratory tests are mostly associated with Kaohsiung (HA), hyperluteinizing hormone (LH), hyperinsulinemia (HI) and insulin resistance (IR), abnormalities of glucose tolerance and lipid metabolism [2].

Approximately half of all PCOS patients are overweight or obese, which is a defining characteristic of the classic type of PCOS known as »obese PCOS« [2]. Women with PCOS have higher rates of infertility, type 2 diabetes, pregnancy-related problems, and hypertension due to their elevated body mass index (BMI). This can lead to adverse metabolic and reproductive outcomes. Although the link between PCOS and obesity is well-established, the precise biological mechanisms behind it remain unclear.

Currently, the treatment of PCOS is focused on symptom alleviation. Oral contraceptives have become the main modality of treatment for PCOS due to their ability to improve hyperandrogenic symptoms, regular menopausal bleeding and prevention of endometrial hyperplasia. In 2013, the Endocrine Society guidelines included metformin as a recommended treatment for addressing endocrine-metabolic disorders and promoting weight loss [3]. According to 2017 guidelines (4), metformin was suggested as a therapy option for promoting ovulation in women with polycystic ovarian syndrome (PCOS) [4].

For morbidly obese patients with obese PCOS, bariatric surgery represents a permanent solution for weight reduction [5]. Studies carried out by Li et al. [6] revealed the efficacy of bariatric surgery for reducing both the incidence of polycystic ovary syndrome and its associated clinical symptoms. While there have been few prospective studies conducted on the subject, numerous retrospective studies have confirmed the significant impact of bariatric surgery on women with both obesity and polycystic ovary syndrome. The benefits include weight loss, restoration of menstrual regularity, and reductions in serum androgens and metabolic dysfunction [7] [8] [9]. Although effective, bariatric surgery is not considered a high-priority intervention for patients with obesity and polycystic ovary syndrome [10]. The effectiveness of medical and surgical treatments has not yet been systematically compared. In this study, we looked at the baseline metabolic characteristics of obese PCOS patients as well as the efficacy of laparoscopic sleeve gastrectomy combined with metformin after 3 months of treatment.

## Materials and methods

### Data source

A total of 104 patients with obese PCOS and obese non-PCOS, aged 20–37 years, were treated in the Department of Gynecology and Minimally Invasive Surgery of the Second Hospital of Hebei Medical University between January and December 2018.

### Diagnostic criteria and grouping

PCOS was judged according to the diagnostic criteriaj revised by ESHRE/ASRM at the Rotterdam meeting in 2003. The 104 patients were divided into obese PCOS group (54 patients) and obese non- PCOS group (51 patients). Based on their treatment methods, the obese patients suffering from polycystic ovary syndrome were split into two groups: one underwent laparoscopic sleeve gastrectomy (comprising 17 cases), while the other was given metformin (comprising 22 cases).

### Statistical analysis

Statistical analysis software statistical product and service solutions (SPSS) 24.0 was used for data analysis. P < 0.05 was statistically significant. Data that followed a normal distribution were presented using mean ± standard deviation and were analyzed using the independent sample t-test to compare between two groups. The 2 test was used for categorical data.

## Results

### Obese patients: A comparison of those with and without polycystic ovary syndrome (PCOS)

104 patients between the ages of 17 and 37 participated in the study. Of these, 53 were obese and had polycystic ovarian syndrome (PCOS), while the remaining 51 were obese but did not have PCOS. According to Table 1, the two groups were matched for age, BMI, waist size, and WHR.

**Table 1:** Comparison between the obese PCOS group and the obese non-PCOS group, for each measurement.

	Obese PCOS group <br>(n=53)	Non-obese PCOS group <br>(n=51)	t	P
Age (years)	26.3±4.5	27.1±5.3	0.808	0.4205
Height (cm)	162.8±5.3	162.4±6.3	0.380	0.7045
Body weight (kg)	88.7±18.4	95.8±22.1	1.792	0.0761
BMI (kg/cm^2^)	33.3±6.4	36.1±7.1	1.856	0.0686
Waist circumference (cm)	104.3±15.0	109.8±16.1	1.824	0.0710
WHR	0.9±0.1	0.9±0.1	0.779	0.4378

### Sex hormone differences

In comparison to the obese non-PCOS group, the obese PCOS group exhibited significantly higher expression of AMH, LH/FSH, T, SHBG, and FAI with P < 0.05 (Table 2). However, FSH, LH, E2, P, and PRL expression did not display obviously differences between two groups (P > 0.05).

**Table 2:** Comparison of AMH and sex hormone indexes in the obese PCOS and obese non-PCOS groups.

	Obese PCOS group <br>(n=53)	Non-obese PCOS group <br>(n=51)	t	P
AMH (ng/mL)	7.6±3.8	4.4±2.4	5.203	<0.0001
FSH (IU/L)	6.0±2.1	5.6±2.0	1.043	0.2993
LH (IU/L)	10.0±4.7	7.8±6.4	1.974	0.0510
LH/FSH	1.7±0.7	1.3±0.8	2.393	0.0186
E2 (pmol/L)	60.4±18.0	62.3±25.3	0.423	0.6662
T (nmol/L)	1.0±1.0	0.6±0.2	5.386	<0.0001
SHBG (nmol/L)	23.3±15.0	29.0±13.5	2.024	0.0456
FAI	18.8±15.2	7.9±5.4	4.813	<0.0001

### Glucose and lipid metabolic indexes

Table 3 shows that the levels of HOMA-IR, TG, LDL, Apo-B, and uric acid were significantly higher in the obese PCOS group compared to the obese non- PCOS group with P < 0.05. Nonetheless, the other variables did not present any substantial variations between the two groups.

**Table 3:** Comparison of glucose and lipid metabolic indexes in the obese PCOS group and the obese non-PCOS group.

	Obese PCOS group (n=53)	Non-obese PCOS group (n=51)	t	P
FPG (mmol/L)	6.3±1.9	5.9±1.7	1.078	0.2835
1hPPG (mmol/L)	10.3±3.3	10.4±3.3	0.240	0.384
2hPPG (mmol/L)	8.6±3.4	8.4±3.7	0.365	0.7158
INS (mmol/L)	28.5±12.6	26.1±19.9	0.686	0.4944
1hINS (mmol/L)	162.1±85.2	143.6±80.9	1.135	0.259
2hINS (mmol/L)	143.6±88.2	116.9±77.6	0.167	0.105
HOMA-IR	8.7±5.1	6.9±5.6	3.169	0.002
HbA1c (%)	6.0±1.2	6.0±1.2	0.119	0.9050
TC (mmol/L)	5.0±0.9	4.3±1.0	1.078	0.2835
TG (mmol/L)	2.1±1.3	1.6±0.7	2.709	0.0079
HDL (mmol/L)	1.1±0.2	1.1±0.3	0.062	0.9510
LDL (mmol/L)	3.4±0.7	2.8±0.7	3.815	0.0002
Apo-B (g/L)	1.1±0.1	1.0±0.2	3.025	0.0031
Uric acid (μmol/L)	398.5±80.3	365.4±64.0	2.316	0.023

### Comparison before and after 3 months of treatment

Effects of bariatric surgery on obese PCOS patients are examined. Weight, BMI, INS, HOMA-IR, and TG were significantly decreased after surgery; the remaining indexes were not statistically changed (Table 4).

**Table 4:** Changes in metabolic indicators after laparoscopic sleeve gastrectomy in obese PCOS patients (17 cases).

	Before surgery (n=17)	3 months after surgery (n=17)	t	P
Body weight (kg)	113.3±25.0	93.7±20.3	2.441	0.021
BMI (kg/cm^2^)	40.7±8.6	33.7±6.9	2.581	0.015
Waist circumference (cm)	117.7±18.2	109.3±16.1	1.400	0.172
Hip circumference (cm)	124.7±16.7	118.4±16.6	1.079	0.289
WHR waist/hip	0.9±0.1	0.9±0.1	0.691	0.495
FPG (mmol/L)	6.5±2.8	5.1±0.8	1.823	0.078
INS (mmol/L)	19.8±11.7	8.3±4.5	3.651	0.001
HOMA-IR	5.8±3.9	1.9±1.2	3.718	0.001
TC (mmol/L)	4.7±0.9	4.8±0.8	-0.548	0.588
TG (mmol/L)	2.1±1.6	1.3±0.4	1.905	0.045
HDL (mmol/L)	1.2±0.5	1.2±0.5	-0.290	0.774
LDL (mmol/L)	3.1±0.7	3.3±0.7	-0.829	0.414
Apo-B (g/L)	1.1±0.2	1.1±0.1	-0.095	0.925

### Analysis of the effect of metformin treatment in obese PCOS patients

Ins, HOMA-IR, and T were significantly decreased, the remaining indicators were not statistically different (Table 5).

**Table 5:** Changes in metabolic indexes of obese PCOS patients (22 cases) taking metformin for 3 months.

	Before drug administration<br>(n=22)	3 months of medication<br>(n=22)	t	P
Body weight (kg)	798±11.4	74.8±11.4	1.441	0.157
BMI (kg/cm^2^)	31.2±4.8	29.2±5.0	1.273	0.210
Waist circumference (cm)	99.0±11.7	95.1±10.9	1.133	0.264
Hip circumference (cm)	103.6±7.4	101.2±10.9	1.108	0.274
WHR waist/hip	0.9±0.1	0.9±0.1	0.798	0.429
FPG (mmol/L)	6.4±1.9	5.6±0.7	1.769	0.085
INS (mmol/L)	27.8±14.5	20.7±8.9	2.455	0.016
HOMA-IR	8.4±6.9	5.2±2.6	1.907	0.044
TC (mmol/L)	4.6±0.6	4.68±0.67	-0.240	0.812
TG (mmol/L)	1.6±0.6	1.8±0.8	-1.093	0.281
HDL (mmol/L)	1.2±0.2	1.3±0.5	-1.077	0.288
LDL (mmol/L)	2.9±0.6	2.9±0.65	0.310	0.292
Apo-B (g/L)	0.9±0.4	1.0±0.2	-1.434	0.159
FSH (IU/L)	5.0±1.6	5.0±1.6	-0.016	0.987
LH (IU/L)	7.9±4.9	6.5±3.8	1.111	0.273
LH/FSH	1.5±0.7	1.3±0.7	0.903	0.372
T (nmol/L)	1.0±0.6	0.7±0.4	2.001	0.038

### Assessing the degree of change in various indices following 3 months of weight reduction surgery and metformin treatment

Table 6 displays the results indicating that, in comparison to the metformin group, the surgery group exhibited a significantly larger decrease in weight, BMI, waist circumference, hip circumference, and TG. However, there were no significant differences in the remaining indicators.

**Table 6:** Comparison of the amount of changes in glycolipid metabolic indexes between the laparoscopic sleeve gastrectomy group and the metformin group.

	Change values before and <br>after surgery (n=16)	Change in value before and <br>after medication (n=22)	t	P
Body weight (kg)	19.6±6.5	5.1±2.3	8.194	<0.001
BMI (kg/cm^2^)	7.1±2.3	1.9±0.8	8.493	<0.001
Waist circumference (cm)	8.5±2.6	3.9±1.7	2.041	0.049
WHR waist/hip	0.02±0.0	0.02±0.0	0.509	0.614
FPG (mmol/L)	1.3±0.2	0.8±0.3	0.766	0.449
INS (mmol/L)	11.4±2.3	7.1±2.7	1.009	0.320
HOMA-IR	3.8±1.0	3.2±1.5	0.356	0.724
TC (mmol/L)	-0.17±0.0	-0.05±0.0	-0.496	0.623
TG (mmol/L)	0.8±0.2	-0.3±0.0	2.921	0.006
HDL (mmol/L)	-0.05±0.0	-0.13±0.0	0.395	0.695
LDL (mmol/L)	-0.2±0.0	0.06±0.0	-1.235	0.225
Apo-B (g/L)	-0.03±0.0	-0.14±0.0	1.413	0.167

## Discussion

Obese PCOS patients with hormonal imbalances. AMH is secreted by granulosa cells with a diameter of less than 8 mm, and AMH levels can be used to estimate the number of early sinus follicles in the ovary [11]. There is evidence to suggest that obese PCOS women display an approximately twofold increase in AMH levels compared to non-PCOS patients. Additionally, these higher AMH levels have been linked to elevated androgen concentrations [12]. Another reason for increased AMH may be insulin, and Glueck et al. [10] observed a direct correlation between AMH and insulin insensitivity. Obesity can cause an increase in ovarian hyperandrogen production by a combination of follicular membrane cell stimulation and ovarian function upregulation. Additionally, obesity has been shown to increase insulin resistance and compensatory hyperinsulinemia, which further promotes adipogenesis while inhibiting lipolysis. As this creates a self-perpetuating cycle, it exacerbates the negative effects of obesity on the body [13]. In comparison to women without PCOS, those with this condition are not only more likely to be obese but also exhibit a higher degree of visceral adiposity index. This increase in visceral adiposity is closely linked to insulin resistance and hyperandrogenism [14]. The results of this study show that levels of AMH, LH/FSH, T, SHBG, and FAI were significantly greater in obese patients with polycystic ovarian syndrome (PCOS) than in obese patients without PCOS. Endocrine abnormalities were also found to be more severe in obese PCOS patients.

The development of polycystic ovary syndrome (PCOS) is highly influenced by insulin resistance (IR). Reported prevalence rates of IR among PCOS patients vary between 14–24% based on ESHRE/ ASRM criteria and 20–43% based on NIH criteria, reflecting differences in PCOS phenotypes and ethnic backgrounds [10]. Compared to women with similar body weights, patients with PCOS are at greater risk for developing IR, which can be further exacerbated by obesity [15]. The current study found HOMA-IR levels to be significantly higher in the obese PCOS group compared to the obese non-PCOS group. IR in PCOS patients can result in high levels of insulin secretion and b-cell function, which can increase their risk of developing impaired glucose tolerance (IGT) and type 2 diabetes mellitus (T2DM). A longitudinal study of women spanning over ten years found that the age-standardized prevalence of diabetes at the end of follow-up was 39.3% among PCOS patients, significantly higher than the prevalence rate of 5.8% reported among healthy women.

Obese patients with PCOS often display disorders in lipid metabolism, which are characterized by an increase in total cholesterol (TC), low-density lipoprotein (LDL) cholesterol, and triglycerides (TG), as well as a decrease in high-density lipoprotein (HDL) cholesterol levels. The presence of obesity can exacerbate these dyslipidemic effects [16]. Di Stolfo et al. [17] found that the prevalence of dyslipidemia in PCOS with obesity was 65% to 81%. Epidemiological studies have revealed a close association between PCOS and cardiovascular disease, with the risk of myocardial infarction in PCOS patients being seven times higher than in age-matched controls [18]. Also an increase in visceral fat plays a crucial role in IR and is an independent risk factor for T2DM [19]. That is, obesity and PCOS together contribute to the development of cardiovascular disease. In addition to its association with cancer-related mortality, elevated serum uric acid (SUA) has been identified as an independent risk factor for cardiovascular events [16]. The current study found that the levels of TG, LDL, Apo-B, and uric acid were all significantly higher in the obese PCOS group compared to the obese non-PCOS group. These findings suggest that obese patients with PCOS may face a higher risk of developing cardiovascular disease.

In addition to reducing serum insulin and androgen levels, metformin has also been demonstrated to promote normal ovulation by eliminating abnormalities in the gonadotropin-releasing hormone (GnRH) cycle and pulsatile production of gonadotropins [20]. Numerous studies have reported that women with PCOS who incorporate metformin into a controlled diet plan experience significant weight loss (usually 8%), lower waist-to-hip ratio, lower IR and T, and improved menstrual cycles. Obese PCOS patients in this study had a significant decrease in INS, HOMA-IR, and T after 3 months of metformin administration. Patients with PCOS and a body mass index (BMI) exceeding 35 kg/m^2^ or at least one comorbidity in conjunction with a BMI greater than 30 kg/m^2^ may be considered for metabolic surgery. It was found that weight loss of 60% to 70% within 2 years after bariatric surgery was associated with a 70% remission rate of T2DM [21]. Overweight and obese patients with PCOS may experience significant benefits from a weight loss of 5–10%, including improvements in various physiological characteristics. The study presented indicates that a small weight loss and decrease in total body fat percentage can positively impact the hormonal profile and restore ovulation in anovulatory obese women. Therefore, it is advised that losing weight be taken into account as a preliminary step before beginning ovulation induction therapy. By generating better results and minimising any dangers or consequences related to medical procedures, this strategy may help patients [22]. However, highly obese patients may require bariatric surgery to achieve a 25–50% weight loss [23]. Bariatric surgery is the recommended initial approach for treating individuals with morbid obesity [24]. Several studies have concluded that bariatric surgery is superior to pharmacotherapy for the control of hyperglycemia in T2DM, with its ability to increase endocrine and glucagon-like peptide-1 (GLP-1), reduce HI, and improve insulin sensitivity [25].

In this study, Insulin resistance was improved in both the drug group and the surgery group, but body weight, BMI and TG were all improved in the surgery group. We already know that obesity, hyperlipidemia, kaohsiong, IR and other factors promote each other, and excess energy may be the root cause of obesity combined with PCOS. Weight loss surgery can block this vicious cycle from multiple dimensions, and can be used in one operation. Continuous weight loss to the ideal weight, and drug treatment requires long-term medication, patient compliance is poor, and easy to rebound. However, bariatric surgery is still underappreciated in the existing guidelines for the treatment of obesity combined with PCOS, and our study suggests that the treatment goal of PCOS combined with obesity should focus on weight loss. Bariatric surgery may be considered especially for obese patients with PCOS who have a poor response to lifestyle interventions and medication. With the increase of people's health awareness, the application of bariatric surgery in this field will have a wide prospect.

The major limitation of our study is that it was originally designed as a randomized trial but failed to be conducted as such because surgical treatment is invasive and differs greatly from drug treatment. In addition, the relatively small sample size and short follow- up time was are also limitations. Furthermore, it is possible that statistical power was inadequate for some comparisons with the small number of patients in the subgroup analysis.

## Dodatak

### Acknowledgements

This work was supported by the Health department project (20230994).

### Conflict of interest statement

All the authors declare that they have no conflict of interest in this work.
